# The Impact of a Rotating Elective on Medical Students' Perception of Radiation Oncology

**DOI:** 10.7759/cureus.7931

**Published:** 2020-05-02

**Authors:** Wyatt MacNevin, Todd Dow, Rumana Rafiq, Margaret Man-Ger Sun, David Bowes

**Affiliations:** 1 Medicine, Dalhousie University, Halifax, CAN; 2 Radiation Oncology, Dalhousie University, Halifax, CAN; 3 Radiation Oncology, Queen Elizabeth II Health Sciences Centre, Halifax, CAN

**Keywords:** radiation oncology, medical students, elective, pre-clerkship residency exploration program (prep)

## Abstract

Background

As Radiation Oncology (RO) is a field with limited exposure in undergraduate medical education curricula, the information sources used to form students' perception of the field can have a substantial impact on whether students decide to pursue experiences in RO. Furthermore, the effects of a single elective experience in RO can strongly influence career decisions as it may serve as the only experience for students to gain an understanding of RO as a specialty. This study analyzes which information sources students use and most strongly value when forming their perception of RO both before and after participating in the program, while also analyzing changes in the perception of various speciality-related factors associated with RO.

Methods

To address underrepresented specialties, the Pre-clerkship Residency Exploration Program (PREP) was developed to provide students exposure to RO and 13 other specialties through half-day clinical rotations, simulations, skills sessions, and panel discussions. A total of 37 participants completed both “Pre-program” and “Post-program” surveys to evaluate which information sources they use and value most when forming their perception of RO, and student perception of career factors associated with RO was assessed.

Results

Students reported that Pre-program information sources of RO were based on Lectures (35 students, 94.6%) and Preceptors (18 students, 48.7%). Post-program responses indicated that the greatest sources of information used were from Preceptors (36 students, 97.3%) and Residents (34 students, 91.9%), with the greatest increase being found in interactions with Residents for gaining specialty information (78% increase). Students most highly valued Preceptors, Residents, and Lectures as information sources when forming their perception of RO. Pre-program, students had the greatest positive perception of RO with respect to Income Potential (mean: 3.76/5.00 ± 0.87), Intellectual Challenge (mean: 3.90/5.00 ± 0.94), and Research Opportunities (mean: 3.86/5.00 ± 0.83) while most negatively assessing the factors of Flexibility (mean: 2.69/5.00 ± 0.93) and Level of Stress (mean: 2.93/5.00 ± 0.94).

Conclusions

Student perception of a medical specialty is a factor that may influence student elective choice and career decisions. Through participating in PREP, significant positive increases were found in students' perception of RO in the areas of Flexibility, Patient Population, Competitiveness of the Specialty, Quality of the Working Environment, and Levels of Stress. This study highlights which information sources students value the most when forming their perception of RO and the impact a single elective experience has on improving student perception of the field. RO-based programs and lectures can be better designed using this information to introduce students to this specialty.

## Introduction

Despite an increasing prevalence of cancer in the population, Radiation Oncology (RO) remains underrepresented in undergraduate medical education [1-4]. This lack of exposure can lead to medical students completing their undergraduate medical education without ever interacting with an RO service or learning about the crucial role radiotherapy plays in cancer management [5-7]. As early clinical exposure and role model identification are factors strongly related to discipline interest in medical students, many students may never have learned about RO as a career option, and training programs may be missing out on recruiting potential candidates [5,8-10].

To provide medical students experience in non-surgical disciplines that are not highlighted in clerkship, the Pre-clerkship Residency Exploration Program (PREP) was created at Dalhousie University in 2018 [9]. PREP, a two-week rotating elective program open to second-year medical students, was designed to maximize exposure to specialities not encountered in clerkship while increasing mentorship opportunities. In the context of RO, PREP served to provide medical students with a half-day elective experience and a residency panel discussion featuring a resident in RO. Previous studies associated with PREP have shown that an elective in RO increases students' interest in the specialty and understanding of the general responsibilities of a radiation oncologist [9]. Because these electives are often the only exposure students have to RO, it is critical to better understand why and how elective experiences affect students’ perceptions of the field and which information sources students use and value most when forming their perceptions.

This study analyzes which information sources students use and most strongly value when forming their perception of RO both before and after participating in the program, while also analyzing changes in the perception of various speciality-related factors associated with RO. The primary objectives of this study are to identify which sources of information students are using and valuing when forming their perception of RO from the perspective of second-year medical students and to examine how this changes after participating in a two-week rotating elective program. The secondary objective of this study is to analyze changes in perceptions associated with a number of factors related to a career in RO after an initial exposure to the field. These objectives are relevant to departments who wish to promote RO to prospective medical students.

## Materials and methods

PREP is a summer elective program created and organized by Dalhousie University medical students. This program is a two-week intensive rotating elective experience available to second-year medical students. As the first two years of the medical school curriculum at Dalhousie University consist primarily of didactic teaching, small group learning, and half day per week electives and clinical skills teaching, PREP serves as the first clinically immersive experience for participants. The program, in its second year, was held during May 27 to June 27, 2019.

Data was obtained from 37 PREP participants selected by lottery from 80 applicants from a second-year medical school class at Dalhousie University. For selection of these students, the only exclusion criterion applied to the applicants was the inability to attend the full two weeks of the program. Participant consent to be involved in this study was voluntary and had no effect on participant inclusion.

All students rotated through half-day electives in RO, Anaesthesia, Pathology, Cardiology, Endocrinology, General Internal Medicine, and Ophthalmology. For the remaining elective slots, students were able to participate in either Adult or Pediatric Hematology, Nephrology or Neurology, and Physiatry or Neonatology and Medical Oncology. Daily one-hour lunchtime talks were planned for students featuring faculty members from Radiology, Rheumatology, Infectious Disease, Pediatric Palliative Care, Geriatrics, Physiatry, and Ophthalmology. A Resident-Life panel discussion was also organized featuring residents from RO, Anaesthesia, Pathology, and Neurology. Students participated in workshops and skill sessions focusing on developing skills commonly used in Anaesthesia, Pathology, and Ophthalmology, as well as ultrasound scanning/interpretation, and procedural skills used in various fields of medicine.

During the students’ elective in RO, they were placed at the Queen Elizabeth II Health Science Centre in Halifax, Nova Scotia. Here they received an orientation session to the specialty, a tour of the treatment facility, and clinical experiences in an outpatient clinic working alongside an attending staff Radiation Oncologist and in some cases with an RO resident [9]. All students had previously received one lecture on RO in the second-year medical school curriculum as part of a two-week oncology unit.

Study participants received three electronic surveys: Pre-program, End of Week 1, and Post-program, distributed via Opinio (ObjectPlanet, Oslo, Norway). Data was collected through a secure web browser. The first (Pre-program) survey was administered and completed on the first morning of the program, prior to the students completing any clinical activities. The second survey was completed by participants immediately following completion of the last session of week 1. The third (Post-program) survey was provided on the last day of PREP immediately following the last scheduled component of the program. The Pre-program and Post-program surveys both assessed the same questions pertaining to sources from where students received the information that shaped their perceptions, the value they place on their information sources, and the students’ perception of all participating disciplines (including RO). Questions pertaining to participant demographics (age, gender, education) were contained within the Pre-program survey. Questions were designed using the Likert scale when assessing student perceptions and the value they place on their information sources, and binary questions (yes/no) were used to assess sources from where students received their information regarding the specialty.

Survey data was exported into the Statistical Package for the Social Sciences (SPSS) software, version 25 (IBM Corp, Armonk, NY), and outcome data was expressed as frequencies and percentages. Data analysis was performed using a Wilcoxon signed-rank test for non-parametric analysis of questions using the Likert scale. For binary questions, paired-sample T-test analysis was used. A 95% confidence interval was used and the significance threshold was set at p = 0.5 for determining statistical significance. One-way analysis of variance (ANOVA) was conducted using a post hoc Bonferroni test to analyze which information sources students value the most.

## Results

Demographics

All the 37 participants (100%) completed both the Pre-program and Post-program surveys. Participant demographics are presented in Table 1, detailing that the majority of participants were female (70.3%) in the age group of 20-24 years (43.2%) and in their second year of medical school (100%). None of the participants indicated that they had a previous elective experience in RO.

**Table 1:** Demographic characteristics of PREP participants (N = 37) PREP, Pre-clerkship Residency Exploration Program

Demographics			
		Frequency	Percentage
Gender	Male	11	29.7%
	Female	26	70.3%
	Undisclosed	0	0.00%
Age (years)	20-24	16	43.2%
	25-26	8	21.6%
	27-28	8	21.6%
	29-30	2	5.41%
	31-32	1	2.70%
	32+	2	5.41%
Year of study	Med - 2	37	100%

Information sources shaping students' perception of RO

**Figure 1:**
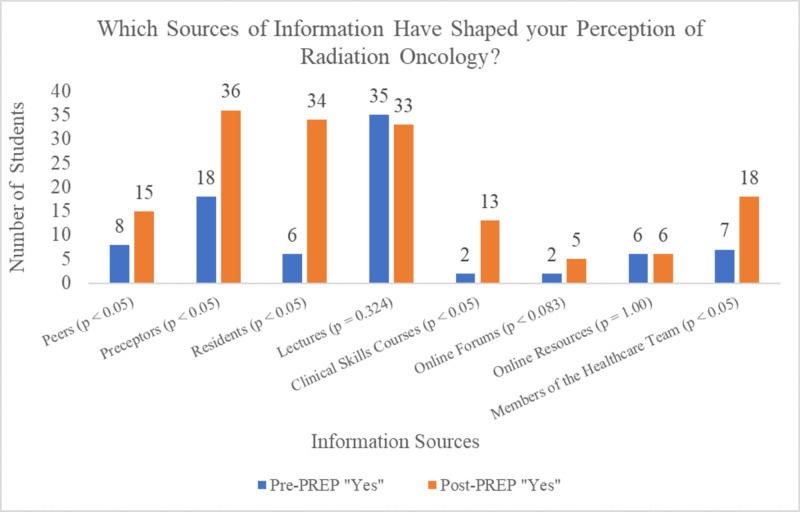
Pre-PREP and Post-PREP responses to which sources of information have shaped students' perceptions of Radiation Oncology, using a yes/no answer structure PREP, Pre-clerkship Residency Exploration Program

Through comparing Pre-PREP and Post-PREP responses, statistically significant increases were found in the number of participants who indicated that Peers, Preceptors, Residents, Clinical Skills Courses (Workshops), and Members of the Healthcare Team had shaped their perception of RO. The greatest increases in information sources used after PREP were for Preceptors and Residents. There were no statistically significant changes found when analyzing the sources such as Lectures, Online Forums, and Online Resource when comparing Pre-PREP and Post-PREP responses. An overview of the data can be seen in Figure 1.

What sources of information do students value the most?

**Figure 2:**
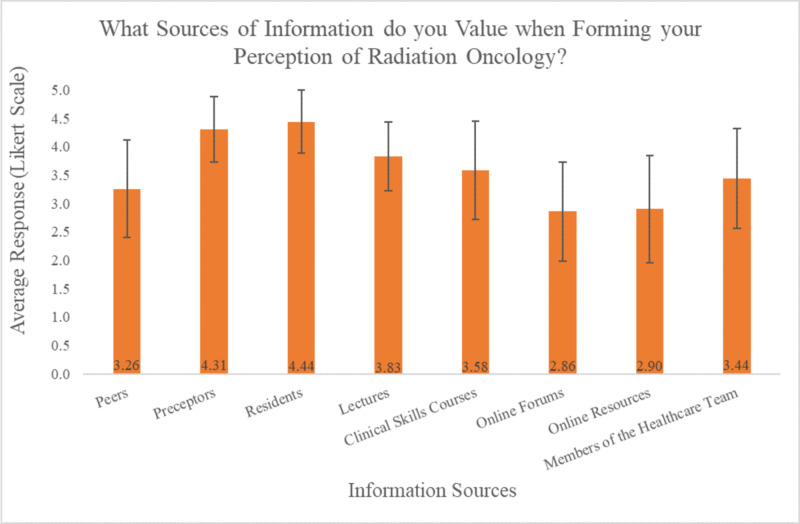
Student responses to "Which sources of information do you value when forming your perception of Radiation Oncology?", using a Likert scale

After identifying which sources of information students use when forming their perspectives on RO, students were asked which sources of information do they value most on both Pre-PREP and Post-PREP surveys. A paired-sample T-test analysis showed no statistically significant differences in the Post-PREP survey regarding how students value the sources of information they receive compared to the Pre-PREP survey. A one-way ANOVA between Post-PREP information sources was conducted to determine the relationship between the information sources that students value. This analysis demonstrated a significant effect for the identified information sources at the p < 0.05 level [F(7, 257) = 19.11, p < 0.001]. Post hoc comparisons using the Bonferroni test indicated that the value of Residents (mean: 4.44 ± 0.56 standard deviation (SD)) was significantly different than Lectures (3.83 ± 0.61), Clinical Skills Courses (3.58 ± 0.867), Members of the Healthcare Team (3.44 ± 0.878), Peers (3.26 ± 0.864), Online Resources (2.90 ± 0.939), and Online Forums (2.86 ± 0.875). Furthermore, this test indicated that the value associated with Preceptors (4.31 ± 0.58) was significantly different from Clinical Skills Courses, Members of the Healthcare Team, Peers, Online Resources, and Online Forums. Lectures were also found to be significantly different from both Online Resources and Online Forums. Additionally, Clinical Skills Courses were found to be significantly different than Online Resources and Online Forums as information sources. These results suggest that students most highly value Residents, Preceptors, and Lectures when forming their perception of RO (Figure 2).

Present perception of RO

Students were asked to evaluate their present perception of RO using a Likert scale with respect to factors associated with the working conditions, opportunities, and lifestyle in the field of RO. The answer option of “cannot assess” was included in the Likert scale response to highlight the effect of PREP on facilitating students to be able to discover certain factors associated with RO.

**Figure 3:**
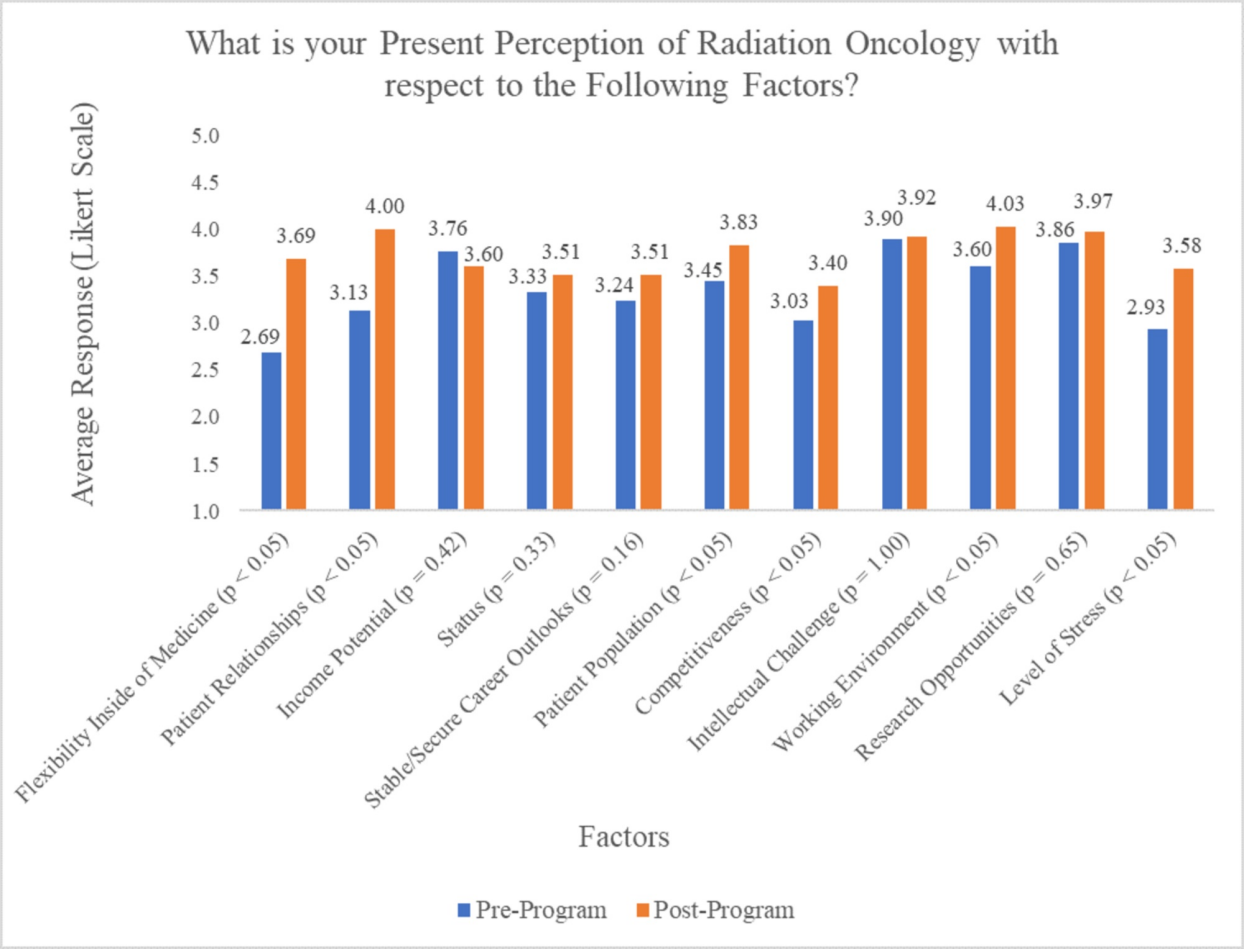
Pre-PREP and Post-PREP responses to students' present perception of Radiation Oncology, using a Likert scale PREP, Pre-clerkship Residency Exploration Program

Before participating in PREP, students acknowledged RO as a field with the favourable factors of Income Potential (mean: 3.76 ± 0.87 SD, p = 0.42), Intellectual Challenge (mean: 3.90 ± 0.94 SD, p = 1.00), and Research Opportunities (mean: 3.86 ± 0.83 SD, p = 0.65). Before PREP, students were most unable to assess the career factors of Flexibility (10, 27.8%), Status (8, 22.9%), and Level of Stress (8, 22.2%). Furthermore, participants most negatively assessed the factors of Flexibility (mean: 2.69 ± 0.93 SD, p = 0.0003), Competitiveness (mean: 3.03 ± 0.89 SD, p = 0.0177), and Level of Stress (mean: 2.93 ± 0.94 SD, p = 0.0096) when evaluating their perceptions of RO (Figure 3).

**Figure 4:**
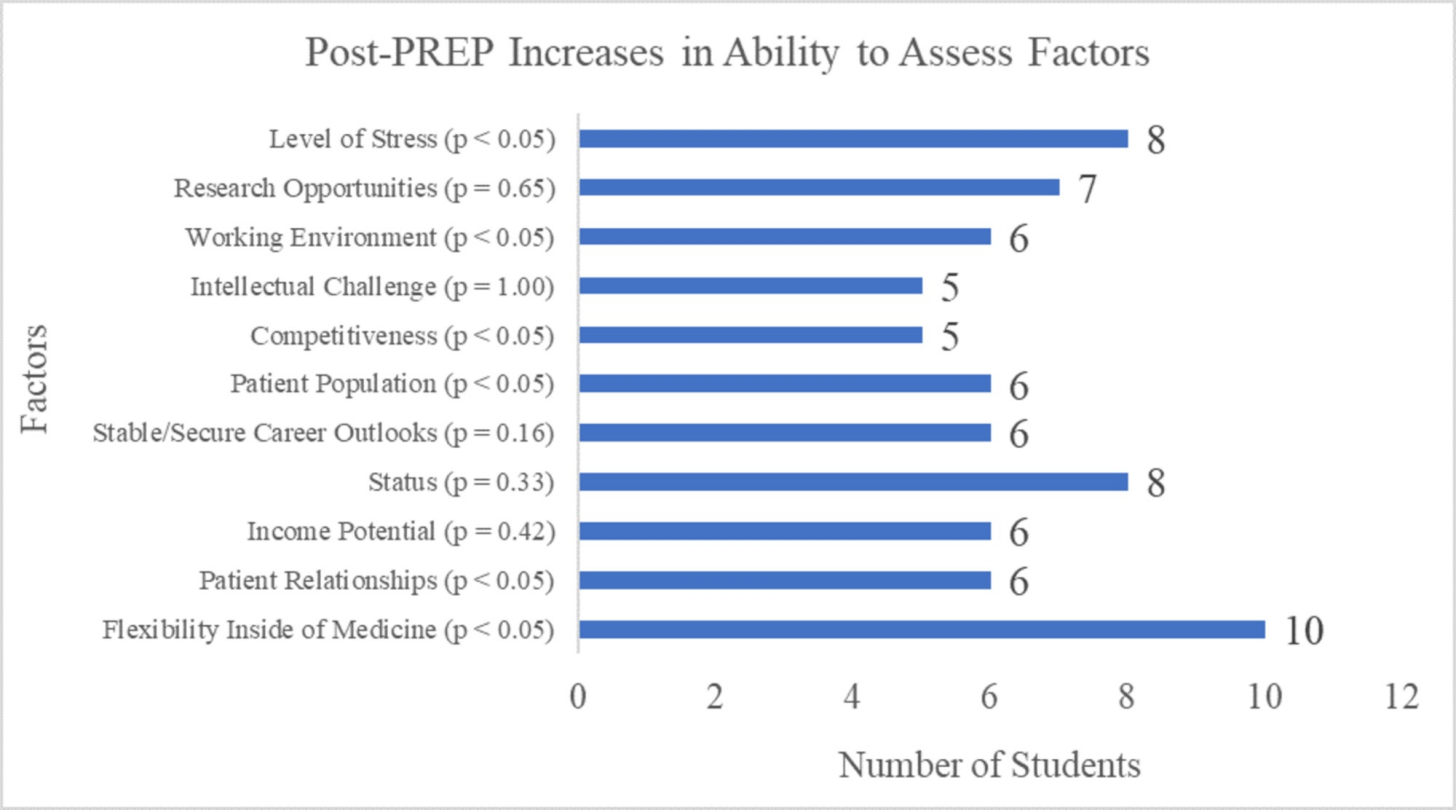
Post-PREP increases in the ability to assess factors associated with Radiation Oncology PREP, Pre-clerkship Residency Exploration Program

Through comparing the number of responses in the Pre-program and Post-program surveys and identifying participants who were “unable to assess” in the Pre-program survey, the greatest increase in the students’ ability to assess the factor was shown in “Flexibility Inside of Medicine” seen in 10 participants (27.78%) (Figure 4).

## Discussion

It is incredibly important and valuable to develop an understanding of student perception of RO both before and after being exposed to the field. This along with a comprehensive understanding of which information sources students use and value most when forming those perceptions is crucial. With this information, early clinical experience opportunities can be better designed and implemented to improve student interest in RO.

This study offers valuable early information as to what information sources students use and value when forming an opinion about RO as a career. Additionally, this demonstrates how students' interest in RO increased after a two-week rotating pre-clerkship elective. As none of the participants indicated that they have had previous elective experience in RO, the outcomes from this study identify the positive effects of a single clinical exposure to RO. As RO is a specialty that medical students are infrequently exposed to, this demonstrates that even a short clinical exposure can have major implications for a student’s career planning, residency preparation, and view of the specialty throughout future clinical experiences [11-13].

Prior to PREP, when students were asked which sources of information were used in shaping their perception of RO, they indicated that the main sources were the lectures they had received in their medical education. The next most common source of information was their various preceptors and peers. As none of the participants had participated in an RO clinical experience prior to PREP, these students would normally only gain perspective of RO through lectures and clinical experiences in associated fields or fields with oncology patients requiring radiation therapy. After participating in PREP, students indicated that their perception of RO was most strongly influenced by information provided by preceptors (100%), residents (94.4%), and members of the healthcare team (50.0%). Students most strongly valued information coming from residents and preceptors, followed by lectures. This relationship demonstrates that students value the information provided by preceptors and residents the most and attribute these sources with having the greatest impact on their perception of the field. Direct exposure to the care team when making career decisions about a particular medical discipline appears to be essential; our data suggests that exposure to a discipline through non-clinical experiences (i.e. lectures) is not as highly valued.

After participating in PREP and gaining exposure to RO, participants had more favourable perceptions associated with the level of flexibility within the specialty, the quality of patient relationships and the favourable patient population. Additionally, students viewed RO as a specialty with a more positive working environment with lower levels of stress compared to their pre-program perception. With this knowledge, RO programs can better focus on communicating more information to prospective students on the flexibility of the speciality (i.e. pathology seen, specialization/fellowship options, rural vs. urban patient care), the positive experiences associated with the unique patient population, and the interdisciplinary and supportive working environments.

We conclude that for students to form their perceptions of RO, it is important to provide opportunities for students to experience RO first-hand where they can be immersed in the speciality while working alongside physicians and residents. When not feasible to provide placement opportunities with RO, it is important to supplement lectures focused on RO or cancer care with information about the specialty such as the flexibility associated with the field and the positive aspects associated with the patient relationships that are formed and the patient population involved within the specialty.

While our study does demonstrate improved student perception of RO following completion of PREP, it does understandably have its own limitations. One limitation of this study is its small sample size and single cohort of data. Additionally, the data presented in this manuscript was collected from one medical school and therefore may not be representative of other institutions. Furthermore, it is not clear how these perceptions would change once students start clerkship; it is conceivable that the types of experiences students have once they are more immersed in the clinical setting may supersede the perceptions students develop through pre-clerkship experiences like we have described here. Also, although all students participated in RO electives at the same location, there was variance between the preceptors and residents present with each student and the type of oncology patient consulted with.

## Conclusions

Student perception of a medical specialty is a factor that may influence student elective choice and career decisions. Through this study, we have analyzed and presented which information sources students utilize and value most when forming a perception of RO and demonstrated that a pre-clerkship elective experience can improve student perceptions of RO as a career. Using this information, RO-based information programs and lectures regarding the specialty can be better designed to introduce students to the specialty and involve them in it.
